# Functional Performance of Plant Proteins

**DOI:** 10.3390/foods11040594

**Published:** 2022-02-18

**Authors:** Kai Kai Ma, Maija Greis, Jiakai Lu, Alissa A. Nolden, David Julian McClements, Amanda J. Kinchla

**Affiliations:** 1 Department of Food Science, University of Massachusetts, Amherst, MA 01003, USA; kkm5269@psu.edu (K.K.M.); mgreis@umass.edu (M.G.); jiakailu@umass.edu (J.L.); anolden@umass.edu (A.A.N.); mcclemen@umass.edu (D.J.M.); 2 Department of Food and Nutrition, University of Helsinki, 00014 Helsinki, Finland

**Keywords:** pulse proteins, legume protein, plant proteins, meat analogs, functional properties, protein isolates, plant-based foods

## Abstract

Increasingly, consumers are moving towards a more plant-based diet. However, some consumers are avoiding common plant proteins such as soy and gluten due to their potential allergenicity. Therefore, alternative protein sources are being explored as functional ingredients in foods, including pea, chickpea, and other legume proteins. The factors affecting the functional performance of plant proteins are outlined, including cultivars, genotypes, extraction and drying methods, protein level, and preparation methods (commercial versus laboratory). Current methods to characterize protein functionality are highlighted, including water and oil holding capacity, protein solubility, emulsifying, foaming, and gelling properties. We propose a series of analytical tests to better predict plant protein performance in foods. Representative applications are discussed to demonstrate how the functional attributes of plant proteins affect the physicochemical properties of plant-based foods. Increasing the protein content of plant protein ingredients enhances their water and oil holding capacity and foaming stability. Industrially produced plant proteins often have lower solubility and worse functionality than laboratory-produced ones due to protein denaturation and aggregation during commercial isolation processes. To better predict the functional performance of plant proteins, it would be useful to use computer modeling approaches, such as quantitative structural activity relationships (QSAR).

## 1. Introduction

Many consumers are shifting toward more sustainable, healthy, and ethical diets, for example, flexitarian, vegetarian, and vegan diets [1]. As a result, the food industry is developing an increasing number of plant-based food products as alternatives to animal-based ones, such as those made from meat, seafood, milk, and eggs. While traditional plant-based protein alternatives (such as tofu, seitan, and tempeh) are commonly accepted among vegetarians and vegans, companies are now creating alternatives, such as meat analogs that are intended to look, taste, and smell more like actual meat products, to increase their acceptability among omnivores [2]. It has been demonstrated that plant-based meat analogues are more acceptable than products produced from less known protein sources such as insects or in vitro cultured “meat” [3,4].

Soybean proteins and wheat gluten are two of the most widely used plant-based ingredients in traditional alternatives to animal products [5]. However, many consumers actively avoid these ingredients. For instance, among households avoiding certain foods or food ingredients in the US, 39% of consumers avoid products containing wheat, particularly due to celiac disease or gluten sensitivity, and 22% avoid products containing soy due to their potential to cause allergic reactions [6]. Although the leading plant protein used in meat alternatives is still soy protein, the percentage of this source fell from around 17% in 2015 to 14% in 2019 among new plant-based products [6]. Pulse proteins derived from leguminous seeds are rapidly emerging as an alternative source of functional plant-based proteins [6]. The availability of a wider range of plant proteins to food formulators had given them greater scope to create plant-based foods with enhanced quality attributes, e.g., non-beany and non-bitter flavor attributes, and therefore enhanced consumer acceptability. Some plant proteins, e.g., soy, contribute to the formation of unappealing flavors from unsaturated fatty acids [7].

Therefore, it is essential to understand the functional properties of these new plant-based proteins and compare them to the more widely used soy and gluten proteins in commercial products and animal-based proteins. The purpose of this paper is to provide an overview of the critical factors impacting the functional properties of plant-based ingredients, with an emphasis on emerging plant proteins such as those isolated from chickpea, pea, lentil, and faba bean. These key factors include cultivars, genotypes, extraction methods, drying methods, protein level, and preparation methods (commercial versus laboratory). In addition, current methods to characterize plant protein functionality are highlighted, including water and oil holding capacity, protein solubility, emulsifying, foaming, and gelling properties, and their limitations are discussed. The impact of the total protein content of emerging plant proteins on their functional performance is also discussed to provide insights into the most suitable ingredient forms to use in different commercial applications. Additionally, we discuss a series of analytical tests to better predict plant protein performance in foods.

## 2. Methods and Search Criteria

By reviewing the available literature, most previous studies have focused on characterizing the functional performance of individual plant proteins, using analytical tools that vary across studies. As a result, it is often difficult to compare the performance of proteins from different sources directly. For this reason, we critically focus on the most common methods currently used to test the functional properties of plant proteins and highlight the ones we believe are most suitable for providing quantitative information that can be compared between studies. Studies highlighting the properties of different legume proteins published from 2007 to 2021 are included. Also, several older articles are included in this review as they propose the original methods to evaluate the functional properties. Articles were searched from Web of Science and Google Scholar using keywords: “pulse protein”, “plant protein”, “plant-based foods”, “legume”, “pea”, “faba bean”, “chickpea”, “lentil”, “soy”, “protein isolate”, “functional properties”, and “extraction methods”, “drying methods”, “gelling properties”, “protein solubility”, “emulsifying properties”, “foaming properties”. Two authors independently searched and selected the papers included in the manuscript. The selection of included articles was done according to the titles and abstracts. Only articles available in English were included.

## 3. Literature Review

### 3.1. Plant Protein Ingredients

Many kinds of plant proteins are available as functional ingredients in foods, including those derived from cereals, legumes, oilseeds, and algae [8]. Among these, pulse proteins are some of the most frequently used because they can be economically isolated from common natural resources (e.g., peas, chickpeas, lentils, and beans) that contain relatively high protein levels (>20 g protein/100 g dry matter), thereby enhancing their economic viability [9]. Extraction and purification methods can convert pulses into functional ingredients with protein contents ranging from relatively low (<50%) to relatively high (>90%), including flour, concentrates, and isolates (Table 1). Many factors affect the functional performance of plant protein ingredients isolated from natural sources, including cultivar type, extraction methods, and drying methods, discussed further below.

#### 3.1.1. Cultivars and Genotypes

Most plant proteins are composed of albumin and globulin fractions [24]. Different cultivars and genotypes naturally have different ratios of these protein components, which influence the functional properties of the extracted plant protein flours, concentrates, and isolates [24]. Indeed, many studies have shown that cultivars and genotypes have a significant impact on the functional performance of plant proteins.

For lentil proteins, the water-soluble fraction reported significantly varied results among cultivars, with red lentil Fırat and green lentil Pul II having the highest contents of around 70 g/100 g [15]. Moreover, past research reports that the proteins in a red lentil concentrate have a higher water-solubility than those in a green lentil one [14]. Lentil proteins have gelling properties that functionally vary among cultivars. For example, the proteins isolated from Ciftci and Kafkas red lentils only form weak gels, even when used at a relatively high concentration (14%). In contrast, those from Ali dayı and Fırat cultivars form hard gels under the same conditions [15]. The oil absorption capacity, foaming capacity, and foaming stability of these proteins were dependent on the cultivar type. Those from Fırat red lentil exhibited the best functional performance. Indeed, these proteins had a higher foaming capacity than soy protein isolate. In another study, Common Blaze red lentil concentrate produced by ultrafiltration had a higher fat absorption capacity than Grandora green lentil concentrate [14].

For chickpea proteins, many researchers have compared the functional performance of those derived from the Kabuli and Desi cultivars. Within the Kabuli type, different cultivars exhibit differences in their water absorption capacity and foaming properties. For instance, Sarı-98 chickpea protein reported a higher water absorption capacity (23%) and foaming capacity (18%) than other cultivars [8]. Another cultivar, Cevdetbey-98, also reported a high water-soluble protein content, good gelation properties, and high oil absorption capacity compared to other cultivars (Canıtez, Gökçe). However, desi and Xena kabuli chickpea cultivars reported similar functional attributes, including water-solubility, water holding capacity, gelation properties, and emulsification properties [14]. In a related study, the functional performance of protein isolates derived from five genotypes of desi chickpea and one genotype of kabuli chickpea were compared [11]. The kabuli chickpea protein isolate had a lower water absorption capacity but higher oil absorption capacity than the desi chickpea protein isolate. These studies suggest that the observed differences may be due to more non-polar amino acids in kabuli chickpea protein, which can help it bind fats. The kabuli chickpea also exhibited the highest foaming stability after 120 min of storage, which may be necessary for some food applications.

Isoelectric points of isolates from different pea protein report similar results being in the range of pH 4.6 to 4.9 [25]. Their water and oil holding capacities were also similar among cultivars. However, the CDC Dundurn isolate had a significantly higher water-solubility (76%) than the other isolates studied (66%), probably due to a lower surface hydrophobicity. Cooper and CDC Dundurn isolates showed significantly lower emulsifying capacity than the other five cultivar isolates, although no significant difference was found among their emulsifying properties. The poor emulsifying performance of CDC Dundurn isolates may be associated with their low surface activity. As a result, this cultivar may not be suitable as an emulsifier in emulsion-based foods.

Moreover, this study suggests a synergistic effect of extraction method and cultivar type on the functional performance of pea proteins, including their water holding capacity, foaming capacity, foaming stability, and emulsifying properties. For example, CDC Meadow isolates had a relatively high-water holding capacity when extracted by salt extraction, but a low one when extracted by micellar precipitation. This demonstrates the importance of optimizing both cultivar-type and extraction method to obtain good functional performance in plant protein ingredients.

Not all pulse proteins show large variations in functionality among their genotypes. For example, several faba bean *Vicia faba* L. genotypes were reported to have similar functional properties [26]. These authors compared the physicochemical properties and functional performance of protein isolates obtained from seven different genotypes of faba bean. The zeta-potential, surface hydrophobicity, protein solubility, oil holding capacity, emulsion capacity, creaming stability, emulsification activity, and stability indices of all genotypes were reported to be quite similar. As the differences in the molecular properties of proteins obtained from different genotypes are relatively small compared to those from different cultivars, there is less concern on which genotype to choose for different food applications. Moreover, the effects of environmental conditions on pea genotype performance have been difficult to isolate [27]. It has also been demonstrated that areas with low rainfall and high temperatures were associated with higher protein content in the same pea genotypes [28].

#### 3.1.2. Different Forms of Plant Proteins

Plant protein ingredients typically come in the form of flours, concentrates, or isolates, depending on their total protein concentrations, typically 50–70%, over 80%, and over 90%, respectively. The more extensive (and expensive) the extraction process used, the higher the protein content in the final ingredient. Protein ingredients also contain different types of proteins, which may have different molecular conformations (native or denatured) and aggregation states (e.g., monomers, dimers, trimers, etc.) depending on their biological origin and the extraction and drying methods used. The concentration, type, conformation, and aggregation state of the proteins in an ingredient play a major role in determining its functionality. In addition, protein ingredients also contain other components that can impact their functional performance, including starches, fibers, lipids, and minerals. Many plant proteins are used as texturized proteins as meat extenders or a meat analog by providing an economical, functional, and high-protein food ingredient [7]. Texturized vegetable proteins are characterized by having a structural integrity and identifiable structure. Obtaining reliable sources of ingredients with the required functional attributes is one of the major challenges in the plant-based food area. In this section, we focus on differences in the behavior of flours, concentrates, and isolates.

Researchers have shown that protein isolates typically have a higher water holding capacity than the corresponding flour form. The higher protein content of isolates attribute to the increase in water holding capacity, while some of the non-protein components in flours may be a barrier to water penetration, such as starch granules, fibers, or lipids. For instance, it was reported that lentil protein isolates had a higher water holding capacity than lentil flours, which was attributed to their lower lipid content and smaller particle size [12]. The oil holding capacity of chickpea protein isolates (2.1 to 4.0 g/g) was reported to be significantly higher than their corresponding flours (1.1 to 1.2 g/g) [11]. Conversely, the gelation properties of great northern bean and chickpea protein isolates were reported to be significantly lower than their corresponding flours [11]. In this case, the gelling properties of the ingredients may depend not only on their total protein content but also on the type, denaturation, and aggregation state of the proteins, and the presence of any non-protein components [29]. The foaming capacity of chickpea protein isolates (30 to 44%) has been reported to be significantly higher than that of their corresponding flours (15 to 20%) [11]. These results suggest that the functional performance of plant proteins is strongly dependent on the protein concentration and form of the ingredients used. Consequently, it is important to identify a protein ingredient that has the molecular, physicochemical, and functional attributes required for the specific application.

#### 3.1.3. Commercial or Laboratory Processed Plant Proteins

Many of the published studies on the functional properties of plant proteins have used isolates or concentrates prepared in a laboratory using small-scale extraction and drying procedures. These studies have provided valuable information about the functional performance of distinct kinds of plant proteins. Nevertheless, there are usually major differences in the functional performance of proteins produced in the laboratory and using commercially viable large-scale processing operations. Commercial processing operations often reduce the functional performance of plant protein ingredients because of protein denaturation, protein aggregation, or the presence of non-protein components that interfere with protein performance.

Similarly, it was reported that the water-solubility of pea protein isolates prepared in the laboratory (66%) was considerably higher than commercial ones (5%) [25]. Several studies have also shown that there are appreciable differences in the water-solubility of commercial soy protein isolates purchased from different sources, which may be due to differences in their biological origin, or the methods used to extract, dry, and store them [15,30]. For instance, although a commercial soy protein isolate and a soy protein extract had similar total protein contents (0.90–0.92 g/g), the water-soluble protein content of the soy protein extract (0.57 g/g) was much higher than that of the commercial protein isolate (0.21 g/g) [15]. This effect might be because the soy proteins were denatured under the highly acidic conditions used in acid precipitation methods, or the higher and longer temperature exposures experienced during drying, which occurs more often in large-scale industrial production [30]. Moreover, other commo practices used in the commercial production of proteins can decrease their solubility and functionality. In addition, plant proteins are often a side product generated during the isolation of edible oils (such as soybean oil), which often involves the use of organic solvents that can denature proteins. In some cases, the functional performance of protein ingredients can be improved by utilizing additional processing operations, such as homogenization or ultrasonic treatment to dissociate aggregates [30].

The nature of the processing operations used to create plant protein isolates has been shown to impact their denaturation state using differential scanning calorimetry (DSC). The thermal denaturation temperature of a laboratory-produced pea protein isolate was reported to be considerably higher (T_d_ = 82.6–94.3 °C) than that of a commercial pea protein isolate (T_d_ = 72.8–72.9 °C) [31]. Moreover, the laboratory-produced pea protein isolate had a much higher transition enthalpy (ΔH_d_ = 15.8–17.8 J/g protein) than the commercial pea protein isolate (ΔH_d_ = 0.033–0.036 J/g protein), which indicates that the commercial ingredient was much more denatured. Furthermore, when the commercial pea protein isolates were heated to about 86 °C, there was a lack of a thermal transition peak, which means that most of the protein isolates were already denatured during processing. As a result, a much higher concentration of the commercial pea protein isolate (14.5%) was required to form a gel than the laboratory-produced version (5.5%). The laboratory-produced pea protein isolates also formed stronger gels than the commercial ones, which was attributed to the higher amount of native protein that could participate in network formation.

It has been reported that commercial soy protein isolates had a higher water holding capacity and lower water-solubility than laboratory-processed ones [32]. Interestingly, when the proteins in the laboratory version were intentionally thermally denatured, the water holding properties of the laboratory-produced and commercial soy protein isolates were similar. This study therefore strongly suggests that protein denaturation is responsible for the poor functional performance of the commercial soy protein isolates. In other studies, commercial soy protein isolates have been shown to have a higher water holding capacity than laboratory-produced ones (7.94 g water/g compared to 1.69 g water/g), but a lower oil holding capacity (1.16 g oil/g compared to 8.23 g oil/g) [15]. The apparent viscosity of commercial soy protein isolates has been reported to be higher than laboratory-produced ones, which may be because some of the proteins have become denatured and aggregated [32].

Overall, these studies demonstrate the importance of carefully characterizing the functional performance of commercial plant protein ingredients from different sources, because they can vary widely. Moreover, the functional performance of laboratory-produced plant proteins reported in the literature may be considerably different from that of commercial plant protein ingredients.

#### 3.1.4. Structure of Plant Proteins

The molecular structure of plant proteins has a major impact on their functional performance. In this section, we therefore briefly review the impact of protein structure on the functional attributes of selected plant proteins. Most plant proteins are primarily comprised of salt-soluble globulins and water-soluble albumins, which occur in a ratio of approximately 70% to 20%, depending on the source of the plant proteins, with the remainder being other minor proteins [24,33]. Legumin (11S) and vicilin (7S) are the main types of globulins found in plant proteins [14]. The most common minor proteins present are convicilin, prolamins, and glutelins [14]. The ratio of legumin and vicilin in plant protein ingredients varies depending on their biological origin and can affect their functional properties. For instance, it was reported that the relatively low legumin-to-vicilin ratio found in pea proteins can increase their functional performance, including their emulsifying and gelling properties, because of their higher protein extractability [34]. Furthermore, it was demonstrated that the amino acid composition, water holding, and oil absorption capacities of red lentil proteins from three origins (USA, Nepal and Turkey) were significantly different [35]. The amino acid compositions of plant proteins also vary depending on the type of species and genotype of the plant used as a source [36]. For example, it was demonstrated differences in essential amino acid content between pea, soy, and lupin proteins: 30%, 27%, and 21%, respectively [37]. In contrast, researchers have demonstrated similar amino acid profiles between faba bean and pea flours, with leucine and lysine found in the highest amount [38,39]. The amino acid composition impacts the functional properties of proteins because it determines the ratio of polar to non-polar groups, as well as the balance of positive, negative, and neutral groups, which affects their surface hydrophobicity and electrostatic interactions. Therefore, the structure of plant proteins is another factor that must be considered when optimizing their functional performance.

#### 3.1.5. Extraction Methods

There are two main categories of protein extraction methods used commercially: dry and wet extraction [40]. These methods are designed to isolate the proteins from the other major components in the original plant material, such as starches, fibers, lipids, and minerals. The most common wet extraction methods include isoelectric precipitation and salt extraction [41]. Isoelectric precipitation (IEP) involves dispersing plant flours in a strong alkaline or acid solution to increase the charge on the protein molecules and thereby solubilize them. The pH of the resulting solution is then adjusted close to the isoelectric point of the proteins to precipitate them. This method is therefore based on reducing the electrostatic repulsion between the protein molecules since they have no net charge around their isoelectric point. Salt extraction (SE) involves dispersing plant flours in a concentrated salt solution, such as ammonium sulfate or sodium chloride solution. At a sufficiently high salt concentration, the proteins that are associated with each other are due to a salting-out effect, which leads to the formation of a protein-rich precipitate phase. Another protein extraction method based on the use of salt is micellar precipitation (MP), but this approach is used to isolate proteins that have a high solubility in concentrated salt solutions but a low solubility in dilute salt solutions [16]. In the MP method, a protein flour is dispersed in a concentrated salt solution to solubilize the proteins (salting-in). This protein solution is then diluted with distilled water to reduce the salt concentration, which results in the association of the protein molecules and the formation of micelle-like protein aggregates that can be removed by centrifugation.

After the precipitated proteins have been collected, they can be re-dispersed within another aqueous solution with a pH and ionic strength that favors high protein solubility. If required, any residual acids, bases, and/or salts used to induce precipitation can be removed by dialysis or filtration [41]. Commercially, ultrafiltration (UF) is often used for this purpose [14]. UF is a type of membrane filtration method that involves applying high hydrostatic pressure to a solution that is in contact with a semipermeable membrane to separate the salts from the protein.

In general, wet processing is an efficient means of carrying out protein extraction, typically leading to samples with a minimum of 70% total protein content [26]. Nevertheless, there are considerable differences in the final protein contents obtained using different wet processing methods for different plant proteins. For instance, chickpea, faba bean, lentil, and pea protein isolates obtained using IEP were reported to have a higher protein content (82% to 88%) than those produced by salt extraction (73–82%) [41]. Soy protein isolates obtained using IEP have also been reported to have a protein content (82% to 86%) that is within this range [18].

The functional properties of plant proteins are highly dependent on the extraction methods used to produce them. For instance, chickpea, faba bean, and pea protein isolates produced by IEP have been reported to have a considerably higher water-solubility than those produced by salt extraction, although lentil and soy protein isolates gave similar values using both methods [41]. In addition, Langton and co-workers used an extraction method called soaked protein extract and IEP for faba beans [42]. The protein content was higher for an alkaline extract (82%) than for a soaked extract (67%). The researchers suggested that the lower protein content in the soaked extract could be due to the absence of a precipitation step resulting in more water-soluble non-protein components, e.g., oligosaccharides in the extract. Moreover, it has been reported that the water holding capacity of pea protein isolates depended on the extraction method used [25]: MP (3.2–3.6 g/g) > IEP (2.4–2.6 g/g) > SE (0.34–2.6 g/g). The authors suggested that the MP method may have exposed more polar groups on the plant protein surfaces, thereby leading to better hydrogen bonding with water. The emulsifying activity of pulse protein isolates produced by IEP has been reported to be significantly higher than those produced by SE [41]. The mean particle size of the oil droplets in emulsions produced by homogenization was reported to be appreciably smaller when the proteins were extracted by IEP rather than by SE [41]. This effect was attributed to the fact that the protein isolates produced by IEP had a slightly higher surface potential and surface hydrophobicity than those produced by SE [25,41]. The surface hydrophobicity of globulins is reported to be higher than that of albumins, which may account for the fact that a higher fraction of globulins is extracted than albumins due to the greater hydrophobic attraction between them [25]. The emulsifying properties, creaming stability, and foam expansion was also reported to be higher for protein isolates produced by IEP than by SE. These results suggest that protein isolates produced by IEP may be better for applications in food emulsions, where small stable oil droplets are often required.

Proteins with improved functional properties can be obtained by using UF alone rather than extraction methods that require pH adjustment or salt addition. For instance, it has been reported that pulse protein concentrates extracted using UF had a slightly higher protein content than those extracted using IEP [14]. The pulse protein concentrates produced by UF were also reported to have higher oil holding capacity and better gelling properties than those produced by IEP. But the water holding capacity, emulsifying properties, and foaming capacity of the protein concentrate were not found to depend on the extraction method. Notably, however, some pulse protein extracts produced by IEP, including green lentil and chickpea concentrates, had higher foaming stability than those produced by UF. This result suggests that both protein type and extraction method should be carefully considered when developing an extraction method for producing plant protein ingredients for specific applications.

Dry processing, also known as air classification, can also be used to produce protein concentrates, but the total protein content is typically relatively low (<50%). In this method, the raw material is ground into a powder and then an air stream is blown through it, which separates the protein and starch fractions based on differences in their particle sizes and densities. For some applications, the dry processing method has advantages over wet processing methods for protein extraction, even though the final protein content is lower. For instance, it has been reported that the final protein content of a faba bean extract was around 64% for air classification but around 90% for IEP [13]. However, the water solubility of the proteins under neutral pH conditions was considerably higher for the air-classified protein (85%) than for the IEP protein (32%). The authors postulated that this difference was due to an increase in the surface hydrophobicity of the faba bean proteins caused by denaturation during the drying process required after wet extraction. Moreover, the air-classified faba bean proteins had a higher foaming capacity and gave a higher gel strength than the IEP ones. The authors suggested that differences in protein denaturation and carbohydrate content in the faba bean protein extracts may have contributed to the observed differences in their functional properties.

Furthermore, other processing methods combined with extraction could change the protein structure and thus influence the functional properties, e.g., heat treatments and extrusion. For instance, black bean protein concentrates treated with high temperature-pressure cooking, showed higher emulsion capacity compared to the raw treatment [43]. Finally, extraction methods can be used individually or in combination to produce a range of protein isolates with different functional attributes from a single flour. Consequently, functional ingredients with properties tailored to specific food applications can be created, e.g., solubility, emulsifying, foaming, or gelling.

#### 3.1.6. Drying Methods

After wet protein extraction, proteins are usually dried to improve their handling and storage stability. Commercially, the most common method used to convert protein solutions into powders is spray drying (SD). This process rapidly converts a liquid into a powder by spraying the liquid into a chamber containing a hot gas, which causes the water to rapidly evaporate. The temperature-time profiles experienced by the proteins must be controlled to avoid excessive protein denaturation. Freeze drying (FD) is more commonly used in research studies to create protein powders from protein solutions on a small scale. The protein solution is frozen and then placed under a vacuum, which converts the ice into a vapor by sublimation, thereby leading to a dried powder. Commercially, spray drying is much more common, as freeze drying is a relatively expensive, time-consuming, low-throughput, and laborious method. Other drying methods have also been developed that use lower processing temperatures to avoid protein denaturation, including vacuum drying (VD) and refractance window drying (RWD). Compared to the other two methods, VD has a faster drying rate, lower drying temperature, and uses an oxygen-deficient processing environment. The drying process used to create a protein powder is known to impact the functional properties of the protein ingredient due to differences in the heating temperatures and times used.

As an example, the functional attributes of lentil protein isolates produced by SD, FD, and VD have been compared [44]. Initially, it was hypothesized that the isolates produced by SD would have the worst functional performance because the temperatures involved in spray drying can reach 80 °C or above. However, the protein isolates produced by SD had a comparable (high) solubility to those produced by FD, which was attributed to the cooling effect associated with water evaporation during spray drying that prevents the temperature of the proteins from becoming too high [45]. In addition, the proteins are only held at high temperatures for a relatively short time in SD. The high solubility of the protein powders produced by SD may also have been due to their smaller and more uniform particle size distribution. Joshi and co-workers showed that lentil protein isolates produced by FD had different functional attributes than those produced by SD. Specifically, the protein isolates produced by VD had significantly lower water solubilities and formed weaker gels, which may have been due to a greater extent of protein denaturation caused by the relatively long drying period (up to 48 h) used. Soy protein isolate produced by VD has also been reported to be more denatured than that produced using other drying methods [46]. VD may also promote protein denaturation when gas bubbles are formed when a vacuum is pulled since proteins are known to adsorb to air-water interfaces and partially unfold, which is known as surface denaturation.

The water-solubility of chickpea protein isolates was found to be appreciably lower when RWD (74.5%) was used to extract them than when FD (94.2%) was used [47]. This effect may have occurred because RWD employs a higher temperature than FD, which may promote more thermal denaturation of the proteins. However, the protein isolates produced by RWD were reported to have a higher water holding capacity than those produced by FD. The RWD protein isolate also had better emulsifying activity than the FD protein isolate, which was attributed to a higher surface hydrophobicity caused by partial protein unfolding. On the other hand, the foaming and gelling properties of the FD protein isolate were better than those of the RWD protein isolate. These results highlight that the drying method used alters the functional performance of protein isolates and should be optimized for specific applications. For instance, RWD protein isolates may be more suitable for application in emulsified foods due to their high surface hydrophobicity and emulsifying activity. In contrast, FD protein isolates may be better in food applications that require good foaming and gelling properties.

### 3.2. Characterization of Plant Protein Functional Properties

In this section, we briefly review several experimental methods that are commonly used to characterize the functional properties of plant proteins.

#### 3.2.1. Water and Oil Holding Properties

The water and oil holding capacities (WHC and OHC) of proteins measure how much water or oil they can hold per unit mass, respectively. These properties are essential for some food applications, such as the syneresis of plant-based yogurts and the cookability and juiciness of plant-based meats. The most common methods used to determine these parameters, which are based on those proposed by [48,49], which involve dispersing a known mass (g/g) of protein in distilled water or vegetable oil followed by vigorous mixing. The resulting slurry is then centrifuged, and the excess water or oil is removed. The difference in the mass of the sample before and after centrifugation is calculated to determine how much water or oil the protein can hold (expressed as g water/g protein or g oil/g protein). It should be noted that this method is not always utilized in a standardized fashion. For instance, different protein concentrations, mixing times, or centrifugation conditions have been used, making it difficult to compare results between different studies. For example, the protein powder takes time to disperse in the surrounding liquid, and the liquid takes time to move into the powder, which means the results depend on mixing time. Moreover, the pH of the protein solution and mixing temperature impact the water retention ability of the proteins. For example, the water retention of soy protein is higher at around pH 6 to 8 and at temperatures from 40 to 70 °C [50]. Although incubation times ranging from 10–30 min did not show any difference in water retention results, a longer incubation time may have an effect. The centrifugation speeds and times used in the analysis also vary in different studies, ranging from 1600 to 16,000× *g* and 10 to 30 min, respectively. It would be advantageous to standardize these conditions, as the measured water holding capacity of protein gels has been reported to increase with increasing centrifugation speed and time. After decanting the supernatant, some researchers have inverted the centrifuge tubes to remove any excess water or oil, leading to some sample losses that impact the results. The WHC may be determined for soluble proteins after the protein solution has been converted into a gelled form, e.g., by heating, cooling, or adding cross-linking agents. The WHC and OHC may also be measured after a protein ingredient has been incorporated into a food product, such as a plant-based meat, fish, egg, or cheese product.

In general, the WHC and OHC values of plant protein isolates increase with increasing protein content (Table 1). However, the extent of the increase is typically different for the WHC and OHC values. For instance, the increase in WHC is more significant than the increase in OHC with increasing pea protein content. The WHC values also depend on plant protein type (soy > chickpea > pea > lentil protein), which may be due to differences in their surface hydrophobicities. WHC and OHC of legume protein isolate range between 1.8–6.8 g/g and 3.5–6.8 g/g, respectively [20,21,51]. Furthermore, the effects of high-pressure processing and heat treatment on the structure and functionality of pulse (lentil, pea, faba bean) proteins were evaluated by [52]. Both treatments resulted in higher water holding capacity for samples compared to untreated controls.

Plant proteins with good water and oil holding capacities are often used as meat extenders or in plant-based meat analogs. For instance, the water holding capacity of beef sausage was improved by adding 2.5% bean flour as an extender, which was quantified by measuring the amount of water the sausage could hold when compressed with a 1 kg weight [53]. It has been reported that the addition of chickpea and pea flour to low-fat pork bologna resulted in a higher cooking yield than the control (less fluid loss), with the chickpea flour giving the best results (>97% yield) [54]. The purge loss, which is the percentage weight loss of the sample after storage, was also significantly reduced after a plant flour was added to bologna. This study showed that the addition of plant proteins helps maintains the fluids within products during storage, which may improve their quality attributes.

Pea protein isolates have been used as meat extenders in chicken nuggets, due to their ability to increase the water holding capacity in a dose-dependent manner [55]. The overall product cook loss also decreased when pea protein isolate was added, decreasing from 12.4% to 5.0%. This effect is due to more water and oil being retained by the plant proteins in the product. Although the cooking loss was lowered, the overall moisture content of the chicken nuggets decreased when more than 3% pea protein isolate was added, which could impact their desirable sensory attributes.

Plant protein concentrates and isolates have also been used as texturized vegetable proteins (TVP) in meat analogs due to their good water holding capacity properties. The WHC influences the porosity and air cell size of the TVPs produced by extrusion [56]. Traditionally, TVPs were made from soy protein isolates, but other proteins are now being utilized for this purpose, including pea, mung bean, and peanut proteins. Pea-based TVP can be produced by high (55%) moisture or low (26–35%) moisture extrusion [57]. Pea protein TVP has been reported to have a higher water holding capacity than mung bean, peanut, and gluten TVP and a higher oil holding capacity than soy and mung bean TVP [56].

A plant protein-based formulation containing a mixture of SPI, gluten, and chickpea flour has been reported to reduce chicken sausage analogs’ cooking loss and shrinkage. These results suggest that plant protein combinations may be used in plant-based meat analogs to improve their water or oil-holding properties.

#### 3.2.2. Gelling Properties

The gelling properties of plant proteins are essential in food applications where a semi-solid structure is required, such as plant-based meat, fish, egg, or cheese products. The most common method for measuring the gelling properties of proteins is based on the determination of the least gelation concentration (LGC), which is the protein concentration where the protein solution forms a gel that does not slide from a test tube after it is inverted [58]. A series of plant protein solutions contains protein concentrations ranging from around 2% to 20%. These solutions are then heated at around 100 °C for a fixed time (e.g., 60 min) to promote thermal denaturation and aggregation of the proteins. After heating, the sample is allowed to cool for a fixed period, and then the tubes are inverted for visual observation. The LGC is the lowest protein concentration where the protein sample remains in the inverted tube. Although this method provides valuable information about the ability of plant proteins to form gels, it does not provide any information about the properties of the gels formed, such as their hardness or brittleness. Therefore, many researchers use additional methods to measure the textural properties of the gels. The most common means of quantifying the textural properties of gels formed from plant proteins is to use compression tests where stress-strain relationships are recorded as a sample is compressed/decompressed at a fixed rate [59]. For example, texture profile analysis (TPA) can measure the hardness, adhesiveness, springiness, cohesiveness, gumminess, and resilience of gels. Using this method, it has been reported that gels formed from lupine proteins had a higher hardness than those formed from pea or faba bean proteins [60]. Dynamic shear rheology measurements can also characterize gel properties, particularly as a function of temperature. For example, Langton and co-workers studied the gelation process of faba bean protein mixtures at pH 5 and 7 as a function of temperature (25 to 95 °C) using dynamic oscillatory measurements [42]. They reported an increase in storage modulus (G’) at a lower temperature, for pH five gels compared with pH seven gels. Other researchers showed that gels formed from kidney bean protein had higher strength and thermal stability than those formed from pea protein [61]. These methods can also determine the gelation temperature and whether a gel is thermally reversible or irreversible.

The gelation properties of plant proteins depend on their nature (Table 1). The least gelation concentration of most plant proteins falls within the range of 10–18%, but some of them can form gels at considerably lower concentrations. For instance, chickpea proteins have a LGC value of around 5–7%. It should be noted that the reported LGC values depend on gelation conditions, such as pH, ionic strength, and heating conditions, as well as on protein type and the presence of other ingredients. Consequently, the same protein may have different LGC values depending on the conditions used, highlighting the importance of standardizing conditions when comparing different protein sources.

Plant proteins are often used as gelling agents to improve the textural attributes of meat products [7]. For instance, it has been reported that the addition of chickpea and lentil flour into beef burgers resulted in a higher hardness [62]. Similarly, adding a chickpea protein concentrate to sausages increased their gel strength [63]. In a different study, it was reported that adding 20% or 60% chicken meat to soy-based sausage did not alter their gel strength or other textural attributes, such as cohesiveness, chewiness, stiffness, adhesiveness, and gumminess [64]. However, the chicken meat-free version of the sausage had a lower gel strength than the hybrid sausages, which may have been due to the higher amount of water in this formulation. Therefore, there is great potential in applying plant proteins in making hybrid meat products to reduce meat consumption and meat-free products.

Researchers have compared the impact of using soy, pea, lentil, and bean proteins as meat extenders in beef patties on their textural properties [65]. They found that the beef patties containing soy protein had the highest hardness, gumminess, and chewiness. The reason that the beef patties containing pulse proteins had lower textural attributes may have been because they had a lower protein content (55–60%) than the soy protein ingredient used (90%). Faba bean flour has been used to create plant protein-based emulsion gels, including yogurt and tofu analogs [66]. Removal of starch from the faba bean flour resulted in a tofu analog with a stronger texture and higher water holding capacity, mainly attributed to increased protein content.

#### 3.2.3. Protein Solubility

Protein solubility impacts various functional properties of plant proteins, especially their emulsification and foaming properties, since it influences their movement to the oil-water or air-water interface [67]. A widely used method for determining the solubility of proteins has been described [68]. In this method, a weighed amount of protein powder is dispersed in a buffer solution, and the pH is adjusted by adding 0.1 M NaOH or HCl. The resulting solution is then stored for a fixed time under standardized conditions, centrifuged, and then the supernatant is collected to evaluate its protein content. Researchers have used slightly different versions of this method. For example, the proteins may be dispersed in the aqueous solution before or after pH adjustment to the final value.

Moreover, the time that the protein is allowed to disperse and dissolve in the aqueous solution varies from 30 min to overnight. Moreover, the stirring conditions and incubation temperature may vary between studies, e.g., refrigerated versus room temperature. For instance, for some plant proteins, an increased solubility was reported when they were incubated at 50 °C rather than 25 °C [67]. In contrast, the influence of laboratory heat treatment (50–100 °C) on pea protein isolate protein solubility was determined by [69]. They concluded that the heat-treated samples had similar protein values as the unheated samples, suggesting that the heat treatment did not cause excessive aggregation that would have produced lower interaction with water. These findings again highlight the importance of standardizing test conditions. For example, it was reported that soybean, faba bean, and pea protein isolates produced by IEP had a higher protein solubility at pH 7 in a study by one group [41] than in a study by another group [17]. This difference might be because the incubation time of the proteins in the solutions was only 30 min in the former study but overnight in the latter study. Finally, the analytical method used to quantify the protein concentration in the supernatant often varies between studies, including the Kjeldahl, Dumas, Bradford assay, and Lowry methods. Consequently, it would be advantageous to use standardized conditions to carry out protein solubility analysis to directly compare results from different studies.

**Figure 1:**
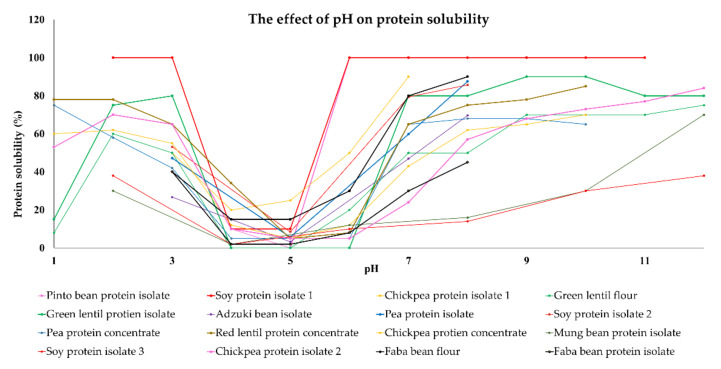
The effect of change in pH on protein solubility of plant proteins reported in published works. Data was extracted from [11,12,13,14,18,47,71,72]. Note: soy and chickpea protein isolate 1,2,3 are reported in different references: [40,47,71,72].

In general, the water-solubility of plant proteins is lowest (<20%) in the pH range from around four to six because their isoelectric points are in this pH range (Figure 1). As a result, there is a relatively low electrostatic repulsion between the protein molecules, which means they can easily associate with each other through van der Waals, hydrophobic, or hydrogen bonding. Conversely, the solubility of plant proteins usually increases when the pH moves away from their isoelectric point because this increases their charge and electrostatic repulsion. For instance, soy, chickpea, faba bean, pea, and lentil proteins have a relatively high water-solubility (>80%) at pH 8, and a moderately high water-solubility (40–60%) at pH 3 (Figure 1). Therefore, it is recommended that pH levels of eight or above are used to optimize protein solubility, but this is not always practical. Meat products like hamburgers and sausages typically have pH values around five to seven depending on the type of meat used, which is close to the isoelectric points of the plant proteins (pH measurement of meat products.). For instance, chorizo sausage containing 3% plant proteins (soy, bean, lentil, or broad bean proteins) as meat extenders had a pH of around 5.8, which is near the isoelectric point of these proteins [70]. In another study, where only plant proteins were used to form a meat analog, the pH was around seven, which meant that the plant proteins were more soluble [64]. It should be noted that it may be beneficial to have both soluble and insoluble proteins in plant-based meat analogs to obtain the desirable textural and other quality attributes.

#### 3.2.4. Emulsifying Properties

The emulsifying properties of proteins are usually characterized by their ability to form and stabilize emulsions [73]. The emulsifying properties of proteins are affected by multiple factors, including the size, shape, flexibility, charge, hydrophobicity, and aggregation state of the protein molecules [74]. Consequently, it is important to have methods to characterize their emulsifying properties.

A variety of methods have been proposed for measuring the emulsifying properties of proteins. Two of the most common approaches were proposed many years ago: the emulsion activity (EA) [73] and the emulsion activity index (EAI) [75]. These methods have been widely used because they can be carried out using simple equipment that is available in many laboratories, although they do have serious limitations. Both methods involve preparing an oil-in-water emulsion by dispersing a weighed amount of protein into a buffer solution and then blending it with a fixed amount of vegetable oil using a high-shear mixer. However, they also have important differences. The EA method involves preparing an oil-in-water emulsion, centrifuging it under controlled conditions (time and speed), and then measuring the volumes of the “emulsion” layer at the top of the tube (V_E_) and the total sample (V_T_) [73]. The emulsion activity is then calculated as EA = 100 × V_E_/V_T_. The EAI method involves preparing a 25% oil-in-water emulsion under standardized blending conditions with a fixed amount of protein in the aqueous phase [75]. A weighed amount of surfactant solution (0.1% SDS) is then added to the emulsion to break up any flocs formed, and then the turbidity of the diluted is measured at 550 nm. An equation is then used to determine the EAI value from the turbidity and droplet concentration (Equation (1)). This method is based on the relationship between the turbidity of an emulsion and the droplet size. As has been pointed out [76], both methods are greatly influenced by the type of blender and blending conditions used in the test, since this leads to emulsions with different droplet sizes, which makes it difficult to compare results between studies. This is because the amount of emulsifier required to stabilize the emulsion depends on both the oil-water interfacial area and the oil concentration. The particle size of the emulsion can be affected by the difference in homogenizing rotational speed as higher speed exerts greater shear forces, which reduces the droplet size [77]. However, these methods are useful for comparing the efficiency of different protein emulsifiers under similar experimental conditions.

(1) $$EAI(\frac{m^{2}}{g})\;=\frac{2\times 2.303\times A_{0}\times DF}{c\times \phi \times \left(1-\theta \right)\times 1000},\;ESI\left(min\right)\;=\frac{A_{0}}{A_{0}-A_{10}}\times 10,$$

Equation (1) shows equations for emulsifying activity index (EAI) and emulsifying stability index (ESI) according to [36].

Where DF is the dilution factor, *c* is the initial concentration of protein (g/mL), *ϕ* is the optical path, *θ* is the fraction of oil used to form the emulsion, and *A*_0_ and *A*_10_ are the absorbance of diluted emulsions at 0 and 10 min, respectively.

Another simple emulsion stability test has been proposed [73]. First, an emulsion is incubated at 80 °C for 30 min to accelerate its breakdown, then it is centrifuged, and then the volume of the cream layer is measured. The emulsion stability is then calculated by measuring the volume of the emulsion layer at the top of the tube after centrifugation: 100 × V_E,H_/V_E,I_, where V_E,H_ and V_E,I_ are the volumes of the emulsion layer with and without the heat treatment. In another proposed emulsion stability test [75], an aliquot of heated emulsion (80 °C for 30 min) is diluted with 0.1% SDS solution and then the turbidity at 550 nm is measured. This method is based on changes in the droplet size of the emulsion after heating, which leads to changes in turbidity. The emulsion activity index and emulsion stability index can then be calculated using the equations shown in Equation (1).

Despite being widely used, the methods just mentioned are overly simplistic and have been superseded by more advanced methods [76]. For instance, the ability of a protein to form emulsions is tested by measuring the mean particle diameter versus protein concentration under standardized homogenization conditions [74]. The minimum protein concentration (C_min_) required to form small droplets can then be established, as well as the minimum droplet diameter that can be achieved (d_min_). In addition, it is sometimes possible to calculate the surface load (Γ) of the emulsifier (mg/m^2^), which determines the amount of emulsifier required to form an emulsion containing a specific droplet concentration and size. The ability of a protein to stabilize an emulsion is determined by incubating the samples under standardized conditions, such as pH (2–8), ionic strength (0 to 500 mM NaCl), temperature (30 to 90 °C) for a fixed period, and then measuring their particle size distribution, microstructure, and creaming stability [78]. In addition, researchers may carry out zeta-potential, surface hydrophobicity, interfacial tension, and rheology measurements to obtain more insights into the performance of plant protein emulsifiers. It should be noted that the use of different methods and operating conditions to determine the emulsifying properties of plant proteins by different researchers makes it difficult to directly compare their functional performance.

The emulsifying properties of plant proteins determined using three of the more traditional methods described above [73,75] are summarized in Table 2. These results show that the emulsifying capacity of plant proteins depends on their origin and concentration. There is great variability between emulsifying activities in different studies although the same EA method has been used [75]. There are, particularly, differences among the same plant protein: emulsifying activity in pea protein isolates varies between 21% and 76%, depending on the study [17,20]. Soybean protein isolate has principally high protein content and EA [20,25], and may therefore be the most suitable for applications where emulsification is important. However, the difference between soy and other plant-based protein is not significant. For example, white lentil protein isolate and cowpea protein isolate also have high EA, 68% and 69% respectively. Table 2b shows there are differences in the emulsifying activity and emulsifying stability of plant proteins between studies, which can be at least partly attributed to differences in the homogenizing speeds and times used. For example, the emulsifying activity index varies again among pea proteins with the smallest reported value of 4.6 m^2^/g and the highest reported value of 42.9 m^2^/g, emphasizing the differences between the used methods.

Surface active plant proteins can be used to emulsify and bind fat in meat products, such as frankfurters and patties. For instance, it has been reported that the addition of lupin flour enhanced the emulsion stability of beef sausage [79]. The quantify of fluids and fats released from the sausages decreased as the amount of lupin flour added was increased, thereby leading to a higher cooking yield. Pulse proteins have also been used as emulsifiers to replace egg yolk in salad dressings [80]. The authors showed that lentil, chickpea, and pea protein isolates could be used to produce salad dressings with similar physical properties as commercial egg-based ones.

#### 3.2.5. Foaming Properties

Plant proteins can also be used to stabilize foams by adsorbing to the air-water interface and creating a protective film around the air bubbles. This is important for food applications, such as cakes, ice cream, and whipped cream, where a creamy or fluffy texture is required. The foaming properties of a protein can be characterized by measuring the foaming capacity and foaming stability. The foaming capacity provides a measure of how much foam a protein solution can create through vigorous mixing, while the foam stability provides a measure of the time a protein takes to stabilize the foam before it collapses. The most common method used to create foams is by whipping a protein solution using a homogenizer or a blender. After the foam is created, the initial volume of the foam is recorded by immediately pouring it into a graduated cylinder, which allows the foaming capacity to be determined. In addition, the change in volume of the foam over time is recorded to assess the foam stability. In different studies, large variations are reported for mixing speeds and times, which again makes it difficult to make direct comparisons among different studies. For example, blending for a longer time or with a higher speed can result in higher foam volume, therefore the calculation for foaming capacity and stability can vary. The foaming capacity can be calculated using the following expression: FC = 100 × (V_2_−_1_)/V_1_, where V_1_ is the volume of protein solution before whipping and V_2_ is the volume of protein solution after whipping. Foaming stability can be calculated as FS = 100 × Vt/V_0_ where V_t_ is the volume of the foam after time t (often taken to be 30 min after whipping) and V_0_ is the initial volume of the foam (immediately after whipping).

The foaming properties of selected plant proteins are reported in Table 3. A wide range of foaming capacities has been reported in different studies, which can be partly attributed to the different blending methods used to create the foams. The highest reported foaming stability was for soy, followed by green lentil, pea, kidney bean proteins, which are all higher than 90%. The higher foaming stability values were reported for proteins that had a relatively high protein content (>90%), suggesting that a greater protein concentration improves foam stability. Some studies have used specific volume (mL/g) as a measurement for foaming properties, which is the ratio of the volume of whipped protein solution to the weight of the whipped solution [81].

Plant proteins exhibiting good foaming properties are often used in baked goods. Lentil protein has been used to replace egg white and milk protein in angel cake and muffins [82]. The final product volume for both muffins and angel cakes did not significantly change when egg white and milk protein were replaced by lentil protein. Moreover, the baked goods containing the lentil protein exhibited lower baking loss than the control. Lupin flour has also been reported to be a potential additive to bread, as the structure and height of the breads did not significantly change after up to 5% substitution of the wheat flour [83].

There is an urgent need to standardize the analytical techniques and protocols used to characterize the functional properties of plant proteins so that comparisons can be made between laboratories. For this reason, we have tabulated a list of proposed methods that can be used for this purpose (Table 4). In the future, it will be important to carry out systematic research to identify the most appropriate analytical methods and protocols for each category of plant-based food (meat, fish, egg, milk, cheese, etc.). Ideally, these methods should be validated and harmonized between different laboratories so that standardized approaches can be adopted in this rapidly emerging area.

## 4. Prediction of Plant Protein Functional Properties

The functional properties of plant proteins are affected by several factors including intrinsic factors such as cultivar type, genotype, and conformation, extrinsic factors such as environmental conditions (pH, ionic strength, sugar content, etc.), and processing conditions (pressure and temperature, etc.) [84]. The application of plant proteins in modern food processing would be facilitated if researchers had better methods to relate the functional properties of these proteins to their molecular and physicochemical attributes. In this section, we therefore briefly discuss some promising approaches that can be used to achieve this goal.

In silico (computer-based) approaches for predicting the functional properties of plant proteins are the same as those used for predicting the functionality of other proteins. These approaches can be conveniently classified into two major groups: (1) statistical-based quantitative structural activity relationship (QSAR) modeling; and, (2) physical-based particle-based simulations.

QSAR modeling aims to develop quantitative expressions that correlate the molecular features of proteins to their functional properties [85]. Models have been developed using a wide range of statistical modeling techniques, ranging from regression methods (such as the partial least square and response surface methods) [86,87,88] to modern machine learning techniques (such as artificial neural networks) [89,90,91].

QSAR models can be predictive even though the underlying biophysical mechanisms are not fully understood. Even so, the quality and availability of the data used to construct the model determine its performance. Therefore, it is of critical importance to develop standard methods to characterize plant protein functionalities, which is currently lacking (see Section 2). The other limitation of QSAR models is that they do not account for the conformational changes of plant proteins under the influence of extrinsic factors such as heat and pressure exposure during extraction and drying processes, which influence their functionalities. The dynamics of protein conformational changes during processing can be resolved by particle-based simulations.

While particle-based simulations include a family of techniques such as coarse-grained, Brownian dynamic, and molecular dynamics simulations, the latter is the most suitable for addressing the length-scale and time-scale of the three-dimensional conformation changes of proteins, such as modeling conformational changes under external processing conditions such as thermal and electric fields [89,92,93,94]. A molecular dynamics simulation involves numerically solving Newton’s equations of motion of all the particles in the system under an applied stimulation. In addition, molecular dynamic simulations are also used in homology modeling in simulating the three-dimensional geometries of proteins based on their amino acid sequences, which can be used as an additional layer of the descriptor in QSAR models [95,96]. We believe that combining physical-based molecular dynamic simulations with statistical-based QSAR models as a hybrid modeling approach will be a promising future trend in predicting the functionalities of plant proteins with higher accuracy and sensitivity.

## 5. Conclusions and Future Work

The number of consumers seeking plant-based alternatives to traditional animal-based foods, such as meat, fish, egg, and milk, continues to grow due to ethical, health, and environmental concerns. As a result, researchers are identifying, isolating, and characterizing a growing number of plant proteins for this purpose. The functional attributes of plant proteins depend on their biological origin, as well as the conditions used to isolate, purify, and dry them. Consequently, specialized ingredients with different applications in the food industry can be developed by better understanding the relationship between their molecular and functional properties. At present, one of the major factors holding back the application of plant-based proteins is the lack of ingredients with consistent and well-defined functional attributes.

Other challenges of developing products completely based on plant ingredients is providing equivalent nutritional composition and mimicking the sensory characteristics. There is growing evidence that suggests alternative plant-based products may not be as healthy as consumers perceive them to be. Several studies comparing the nutritional composition of alternative products conclude significant differences in macronutrients, vitamins, and minerals (e.g., [97,98]). Diets consisting of meat and dairy analogs may result in nutritional deficiencies when compared to an omnivore diet [99]. Opportunities to improve the nutritional quality include making changes to the composition, improving the bioavailability which is low due to the naturally occurring antinutrients present in plants, either through modifications in processing or fortification [97,98]. Sensory and consumer acceptance remains a high priority for these food products, however, there are few studies with sensory evaluation as the primary objective. Recent reviews summarize the existing understanding of the sensory perception of plant-based meat [100] and cheese [101] and suggest ways of improving the sensory methodology and rigor in evaluating plant-based foods.

Ingredient functionality often varies from batch to batch due to changes in biological origin, isolation conditions, or processing conditions. Often, the proteins are denatured and/or aggregated during the production process, which negatively impacts their performance and reduces consumer acceptance. Moreover, plant protein ingredients may contain various kinds of impurities that impact their performance in an unknown way. In the future, it will therefore be important to develop more consistent and gentle processing operations to produce high-quality plant protein ingredients for specific applications. Another factor holding back the development of plant proteins is the lack of standardized methods to characterize their functional properties. Different researchers use different analytical methods, which makes direct comparison of the results on plant proteins challenging. In the future, it will therefore be important to develop a suite of standardized methods that all researchers can use to compare plant proteins under similar conditions. This will enable the best plant protein candidates to be selected for particular applications. Finally, more research is required to establish a better understanding of the relationship between the molecular and functional attributes of plant proteins, as this would allow food manufacturers to identify and select proteins that can produce the functionality required in a particular application. Important advances are being made in this area using modern computational methods. Finally, any plant-based food product must have the desirable sensorial and nutritional properties, which requires the utilization of appropriate sensory methods and an understanding of their gastrointestinal fate.

## Figures and Tables

**Table 1:** Water and oil holding capacity (WHC and OHC) and least gelation concentration (LGC) of plant proteins presented in order of overall protein concentration as reported by the respective publication source. All reported in dry basis except *, which are not reported in literature or reported in wet basis.

Forms of Protein	Protein Type	Protein Content * (%)	WHC (gH2O/g)	OHC (g oil/g)	LGC (%)	References
Flour	Chickpea (desi)	20.0	2.20	1.15	N/A	[10]
	Chickpea	20.6–26.7	1.40–1.50	1.05–1.24	10–14	[11]
	Chickpea (Kabuli)	26.9	1.92	1.25	N/A	[10]
	Green lentil	27.3	1.00	1.70	N/A	[12]
	Faba bean (protein rich flour)	64.1	N/A	N/A	7	[13]
Concentrates	Chickpea	63.9–76.5	2.50–3.10	1.20–1.40	10–14	[14]
	Soybean	70.0	4.52	1.73	>14	[15]
	Chickpea	71.0–77.0	4.90–7.94	10.9–14.6	5–7	[15]
	Red lentil	78.2–82.7	3.70–4.10	1.10–2.30	10–12	[14]
	Green lentil	79.1–88.6	3.40–3.90	1.20–1.35	8–12	[14]
	Pea	80.6–89.0	1.91–2.37	1.10–1.40	N/A	[16]
	Faba bean	81.2	1.80	1.60	14	[17]
	Mung bean	81.5	3.33	3.00	12	[18]
	Pea	81.7–83.9	3.90–4.50	1.20–1.75	12–14	[14]
	Soybean	82.2	1.30	1.10	16	[17]
	Pea	83.6	1.52	1.40	18	[19] *
	Pea	84.9	1.70	1.20	18	[17]
	Mung bean	85.5	1.63	1.13	16	[19] *
	Soybean	86.0	3.0	3.45	14	[18]
Isolates	Lentil	N/A	6.78	6.37	N/A	[20]
	Cowpea	N/A	6.08	5.83	N/A	[20]
	Faba bean	N/A	6.52	5.09	N/A	[20]
	Chickpea	N/A	5.44	5.37	N/A	[20]
	Soybean	N/A	2.39	5.37	N/A	[20]
	Runner bean	N/A	5.43	3.46	N/A	[20]
	Bean	N/A	5.43	5.59	N/A	[20]
	Pea	N/A	6.00	4.84	N/A	[20]
	Akkus bean	N/A	1.9	4.1	9	[21]
	Gembos bean	N/A	1.9	4.0	10	[21]
	Simav bean	N/A	1.8	5.4	9	[21]
	Hinis bean	N/A	2.1	4.7	9	[21]
	Bombay bean	N/A	2.0	4.0	8	[21]
	Different bean	80.8–84.4	1.8–2.1	4.0–5.4	N/A	[21]
	Green mung bean	84.7	2.2	1.76	16	[22]
	Pigeon pea	86.9	3.6	1.16	8	[22]
	Grass pea	87.50	2.15	1.19	N/A	[23]
	Yellow lentil	87.8	1.2	1.78	14	[22]
	Commercial soy	88.6	1.5	0.89	20	[22]
	Chickpea	89.1	2.3	1.73	12	[22]
	Pea	89.2	3.5	1.75	16	[22]
	Yellow mung bean	90.0	2.2	1.72	15	[22]
	Faba bean	90.1	N/A	N/A	12	[13]
	Cowpea	91.0	2.8	1.44	13	[22]
	White lentil	91.2	4.9	1.80	11	[22]
	Chickpea (Kabuli)	91.49–98.65	3.48–3.95	3.65–4.45	N/A	[10]
	Soy	92.4	1.5	1.16	10	[22]
	Grass pea	92.5	2.70	1.37	N/A	[23]
	Chickpea (Desi)	92.7–96.4	2.62–3.78	3.24–4.14	N/A	[10]

N/A = not available.

**Table 2:** The emulsifying property of plant proteins reported in published works using different methods. (**a**) The emulsifying activity (%) is the ratio of the height of the emulsified layer to the height of total contents in the tube, and the emulsifying stability (%) is the ratio of the height of emulsified layer after being heated at 80 °C for 30 min to the height of the emulsified layer before heating. (**b**) Pearce and Kinsella’s method of emulsifying activity index and emulsifying stability index.

(a)				
Protein Type	Protein * Content (%)	Emulsifying Activity (%)	Emulsifying Stability (%)	References
Mungbean protein isolate	81.5	63.2	62.8	[18]
Pea protein isolate	83.6	21.0	43.2	[19]
Green mung bean protein isolate	84.7	62.0	53.0	[22]
Mungbean protein isolate	85.5	41.1	45.5	[19]
Soybean protein isolate	86.0	74.5	81.2	[18]
Pigeon pea protein isolate	86.9	73.0	71.0	[22]
Grass pea protein isolate	87.5	87.5	29.8	[23]
Yellow lentil protein isolate	87.8	72.0	64.0	[22]
Commercial soy protein isolate	88.6	54.0	49.0	[22]
Chickpea protein isolate	89.1	66.0	53.0	[22]
Pea protein isolate	89.2	76.0	62.0	[22]
Yellow mung bean protein isolate	90.0	62.0	53.0	[22]
Cowpea protein isolate	91.0	69.0	61.0	[22]
White lentil protein isolate	91.2	68.0	67.0	[22]
Soy protein isolate	92.4	71.0	70.0	[22]
Grass pea protein isolate	92.5	35.8	28.7	[23]
**(b)**				
**Protein Type**	Protein Content * (%)	**Emulsifying Activity Index (m^2^/g)**	**Emulsifying Stability Index (Min)**	**Reference**
Akkus bean	N/A	22.0	164.2	[21]
Gembos bean	N/A	19.9	60.1	[21]
Simav bean	N/A	21.6	135.4	[21]
Hinis bean	N/A	15.6	60.5	[21]
Bombay bean	N/A	19.6	62.3	[21]
Chickpea	63.9–76.5	5.7	19.70	[14]
Soybean	72.6–87.6	43.4–44.2	25.0–86.0	[41]
Green lentil	74.7–81.9	37.2–44.5	11.0–86.8	[41]
Red lentil	78.2–82.7	5.1	19.2	[14]
Green lentil	79.1–88.6	5.0	17.8	[14]
Pea	80.6–89.0	31.1–39.1	11.0–11.3	[16]
Pea	81.1–88.8	42.73–42.9	10.9–12.4	[41]
Chickpea	81.6–85.4	33.8–47.9	10.9–82.9	[41]
Faba	82.0–84.1	37.1–44.3	11.0–62.4	[41]
Pea	84.90	4.6	18.0	[14]
Kidney bean	90.8–94.7	21.3	46.0	[61]
Kidney bean	92.5	23.7	30.9	[30]
Pea	92.8	13.1	78.1	[61]

* All reported on dry basis except [18,19] are not reported while [30,41] are reported on wet basis. N/A = not available.

**Table 3:** The foaming property of plant proteins reported in published works using different methods. All reported on a dry basis except *, which are not reported, while [30] is reported on a wet basis. ^1^ Foaming capacity was expressed as the volume (%) increase due to whipping. ^2^ Foaming stability was expressed as the ratio of foam volume after 30 min times and the initial volume. ^#^ Calculated according to the reported initial foam volume and foam volume after standing for 30 min.

	Protein Type	Protein Content * (%)	Foaming Capacity or Expansion ^1^ (%)	Foaming Stability ^2^ (%)	Reference
Flour	Soybean	70.00 *	32.0 ^#^	43.7 ^#^	[15]
	Chickpea	71.00–77.00 *	43.9 ^#^	64.8 ^#^	[15]
Consentrates	Faba bean	81.2	15.0	77.0	[17]
	Mungbean	81.53 *	89.7	78.3	[18]
	Soybean	82.20	22	93	[17]
	Pea	83.60	78	N/A	[19]
	Pea	84.90	15.0	94.0	[17]
	Mungbean	85.46 *	110.0	N/A	[19]
	Soybean	86.00 *	68.7	100.0	[18]
	Green Lentil	87.00–95.00 *	34.8 ^#^	96.7 ^#^	[15]
Isolate	Akkus bean	N/A	91	72	[21]
	Gembos bean	N/A	76	82	[21]
	Simav bean	N/A	81	71	[21]
	Hinis bean	N/A	72	80	[21]
	Bombay bean	N/A	83	75	[21]
	Pea	80.60–89.00 *	81.1 *	27.1 *	[25]
	Grass pea (optimized extraction yield)	87.50	87	78	[23]
	Chickpea	89.90–94.40	30.4–44.3	N/A	[11]
	Soybean	90.00 *	24.0 ^#^	66.7 ^#^	[15]
	Soybean	92.00 *	36.0 ^#^	88.9 ^#^	[15]
	Faba bean	92.14–99.36	143.3–183.3	55.9–71.59	[26]
	Kidney bean	92.5	244.9	87.8	[30]
	Pea	92.8	87.0–132.0	94.0–96.0	[61]
	Grass pea (optimized protein content)	92.5	41	100	[23]

N/A = not available.

**Table 4:** The proposed standardized factors for measuring important functional properties of plant proteins.

Functionality	Proposed Standardized Factors
Water/Oil Holding Capacity	Protein concentration, mixing method (incubation time, temperature, pH), centrifugation time/speed
Gelling property	(1) Lowest gelation concentration: mixing method (dissolution time, temperature, pH), heating conditions (time, temperature); (2) Rheology: texture analysis (compression speed, strain amount, etc.)
Protein solubility	Mixing method (dissolution time, temperature), solution conditions (pH, buffer type, ionic strength)
Emulsifying property	Homogenizer operating conditions (such as pressure, number of passes, and temperature), protein concentration, oil-water ratio
Foaming property	Blender rotational speed/time, protein concentration, solution conditions (pH, buffer type, ionic strength), temperature
